# Scientific production of Brazilian speech language pathologists in sleep medicine

**DOI:** 10.5935/1984-0063.20180033

**Published:** 2018

**Authors:** Camila de Castro Corrêa, Fabiane Kayamori, Silke Anna Theresa Weber, Esther Mandelbaum Gonçalves Bianchini

**Affiliations:** 1 Botucatu Medical School, UNESP, Department of Ophtalmology, Otolaryngology and Head and Neck Surgery - Botucatu - São Paulo - Brazil.; 2 Pontifícia Universidade Católica, Department of Speech-Language Pathology - São Paulo - São Paulo - Brazil.

**Keywords:** Speech, Language, Hearing, Sleep Disorders, Intrinsic, Sleep Apnea, Obstructive

## Abstract

**Introduction:**

Previous diagnosis and intervention in patients with sleep-disordered breathing involves several health professionals. Speech-Language and Hearing Sciences (SLHS) performance has been solidified through scientific production.

**Objective:**

To describe the inclusion of Brazilian Speech-Language Pathologists (SLP) in the field of sleep disorders, through the description of studies, scientific publications and participation in scientific events.

**Data Synthesis:**

A search and an analysis of the Brazilian SLP publications in the field of sleep disorders were carried out, including articles, monographs, dissertations, thesis and abstracts published in annals of events. The databases Lilacs, SciELO, Pubmed, Google Scholar tool and Lattes platform were accessed, with final search in January 2018. The analysis consisted of a description of the year of publication, type of publication, area of the SLHS, place of publication and/or event. 40 articles were found in national and international journals, from 1999 to 2017. In relation to publications in books, one book about the subject was published in 2009 and eight chapters of books were published. In the monograph format, 21 studies were carried out, there are 13 dissertations and eight thesis. A total of 151 abstracts were published in annals of scientific events, from 2001 to 2017 and 63 lectures were conducted by SLP.

**Conclusion:**

The inclusion of Brazilian SLP in the area of sleep disorders has been supported by scientific publications in the format of articles in national and international journals, monographs, thesis, dissertations, books and publications in event annals.

## INTRODUCTION

Sleep disorders are frequent problems and compromise the quality of life and general health of affected patients^1^^-^^3^. Because they are associated with different etiological factors and consequences, the diagnosis and treatment of sleep disorders require interdisciplinary teams^4^. The inclusion of the Speech-Language Pathologists’ (SLP) approach, specifically related to sleep-disordered breathing, has been recommended by several scientific articles, Brazilian SLPs being pioneers in this area.

In 2009, the first Brazilian study for which an SLP was part of the team and led a treatment, a randomized clinical trial, was published in an international scientific journal. The authors demonstrated the effectiveness of oropharyngeal exercises in patients with moderate obstructive sleep apnea (OSA)^5^. However, their research started earlier, the authors publishing a monograph in 1999 and an abstract in a national interdisciplinary event in 2001^6^^,^^7^.

Since then, the Brazilian scientific pioneering in the field of sleep disorders has been solidified, with an increasing number of publications of articles, abstracts in annals of scientific events, and lectures given at national and international events^8^.

These publications are essential to disseminate the diagnosis and treatment modalities of SLPs and reinforce the interface with other professionals of the multi-professional team, such as physicians and dentists^9^. The professional activities of SLPs solidify their interactions as being more assertive in the area of diagnosis and treatment of sleep-disordered breathing^9^.

Thus, the objective of the present study was to describe the inclusion of Brazilian Speech-Language and Hearing Science (SLHS) in the field of sleep disorders, by the review of studies and scientific publications, as well as participation in scientific events.

## REVIEW OF LITERATURE

The authors conducted a survey and analysis of the publications of Brazilian SLPs, without limits of time, in the field of sleep disorders, including articles, monographs, dissertations, thesis, abstracts published in the annals of events, and lectures.

As a search strategy of scientific articles, the authors assessed the interfaces of the Lilacs, SciELO (The Scientific Electronic Library Online), PubMed, and Google Scholar (referring to the first five pages containing 10 results each), using the following descriptors and free terms:

·*1^st^ search strategy* : “speech pathology” AND “apnea”;
·*2^nd^ search strategy* : “speech pathology” AND “sleep disorders”;
·*3^rd^ search strategy* : “speech pathology” AND “snoring”;
·*4^th^ search strategy* : “oropharyngeal exercises” AND “apnea”;
·*5^th^ search strategy* : “oropharyngeal exercises” AND “sleep disorders”;
·*6^th^ search strategy* : “oropharyngeal exercises” AND “snoring.”
·*7^th^ search strategy* : “speech therapy” AND “apnea”;
·*8^th^ search strategy* : “speech therapy” AND “sleep disorders”;
·*9^th^ search strategy* : “speech therapy” AND “snoring.”

In addition, the authors reviewed the references of the articles to include articles that were missed using the search strategy.

For the inclusion of abstracts published in annals and presentations in events, the authors performed a manual search on the Lattes platform-CAPES of the authors’ names for the included articles. The coauthors were also considered, and their names were included in the search on the Lattes platform.

As inclusion criteria, the authors considered studies that included a Brazilian SLP in the publication team and that addressed all kinds of interactions of SLPs with patients with sleep disorders, from health promotion to assessment and treatment, with no language restriction. Articles not relevant to the proposed theme, as well as those for which all authors were from countries other than Brazil, were excluded.

The analysis consisted of the description of the year of publication, type of publication, area of SLHS, and locale of publication and/or event.

The last access was in January 2018.

## RESULTS

**Figure 1:**
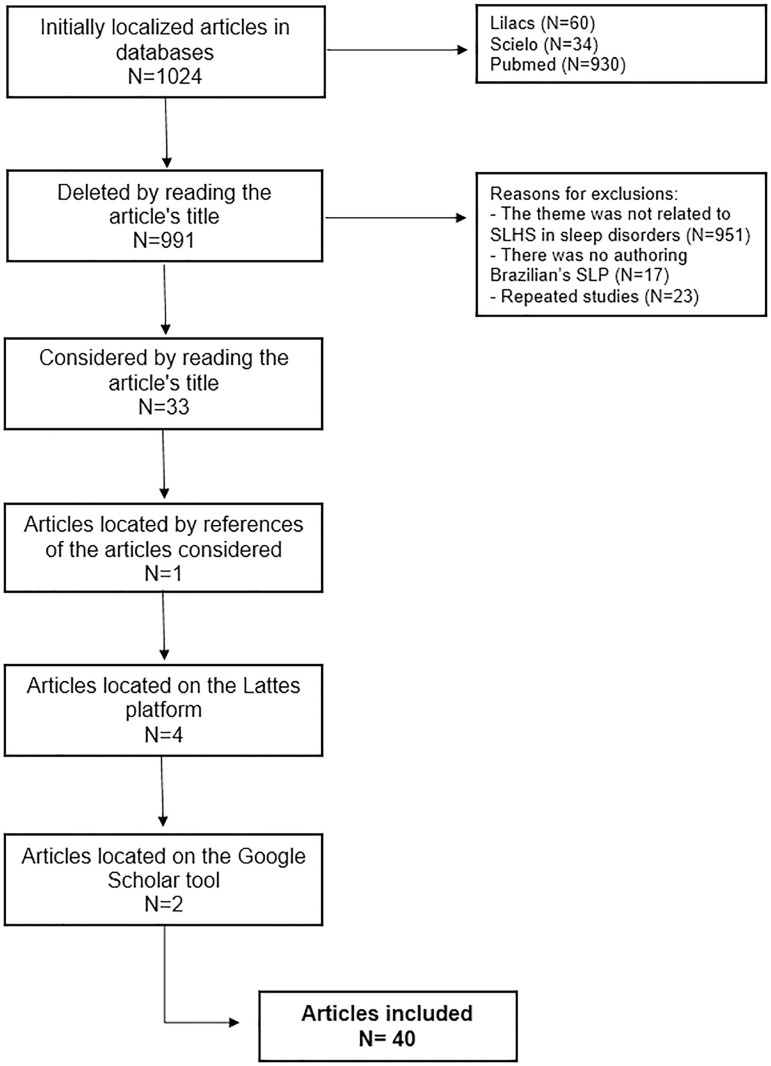
Flowchart of the article selection process.

Of the articles that were selected (Table 1 and Figure 1), 40 articles were published in national and international journals during the period of 1999 to 2017. Table 2 classifies this material by year of publication, title, and journal.

**Table 1:** Results of search strategies in Lilacs, SciELO, and PubMed.

	LILACS	SCIELO	PUBMED
1^ST^	8	3	73
2^ND^	4	4	116
3^RD^	7	3	26
4^TH^	1	1	17
5^TH^	0	1	10
6^TH^	1	1	10
7^TH^	16	9	235
8^TH^	12	4	349
9^TH^	11	8	94

**Table 2:** Scientific articles by Brazilian SLPs about sleep disorders, classified by year of publication, title, and journal.

Year	Title	Journal
2017	*Effects of orofacial myofunctional therapy on the symptoms and physiological parameters of sleep breathing disorders in adults: a systematic review^10^*	CEFAC Journal [Portuguese]
2017	*Obstructive sleep apnea and oral language disorders^11^*	Brazilian Journal of Otorhinolaryngology
2017	*Sleep quality and communication aspects in children^12^*	International Journal of Pediatric Otorhinolaryngology
2017	*Sleep quality in children: questionnaires available in Brazil^13^*	Sleep Science
2017	*Sleep quality in medical students: a comparison across the various phases of the medical course^14^*	Brazilian Journal of Pulmonology
2016	*Different craniofacial characteristics predict upper airway collapsibility in japanese-brazilian and white men^15^*	Chest (American College of Chest Physicians)
2016	*Educational program applied to obstructive sleep apnea^16^*	Journal of Communication Disorders, Deaf Studies & Hearing Aids
2016	*Myofunctional therapy improves adherence to continuous positive airway pressure treatment^17^*	Sleep & Breathing
2016	*Orofacial motor functions in pediatric obstructive sleep apnea and implications for myofunctional therapy^18^*	International Journal of Pediatric Otorhinolaryngology
2016	*Profile of scientific production of obstructive sleep apnea in interface of speech and language pathology^19^*	CEFAC Journal [Portuguese]
2016	*Upper airway collapsibility assessed by negative expiratory pressure while awake is associated with upper airway anatomy20*	Journal of Clinical Sleep Medicine
2015	*Effects of oropharyngeal exercises on snoring^21^*	Chest (American College of Chest Physicians)
2015	*Health promotion in obstructive sleep apnea syndrome^22^*	International Archives of Otorhinolaryngology
2015	*Myofunctional therapy applied to upper airway resistance syndrome: a case report^23^*	CoDAS
2015	*The relation between the apnea and hypopnea on sleeping obstruction, oral breathing and obesity enphasing on speech and voice therapy: a bibliographic study^24^*	Journal of Communication Disorders [Portuguese]
2015	*Validity and reliability of a protocol of orofacial myofunctional evaluation for patients with obstructive sleep apnea^25^*	European Journal of Oral Sciences
2014	*Model of oronasal rehabilitation in children with obstructive sleep apnea syndrome undergoing rapid maxillary expansion: research review^26^*	Sleep Science
2014	*Obstructive sleep apnea and dysphagia: case report^27^*	PET Informs (FOB / USP) [Portuguese]
2014	*Perception of the bed partner and the individual suffering from snoring/osas before and after speech therapy^28^*	CEFAC Journal [Portuguese]
2014	*Upper airway collapsibility is associated with obesity and hyoid position^29^*	Sleep
2013	*Effect of speech therapy as adjunct treatment to continuous positive airway pressure on the quality of life of patients with obstructive sleep apnea^30^*	Sleep Medicine
2013	*Predictors of uvulopalatopharyngoplasty success in the treatment of obstructive sleep apnea syndrome^31^*	Sleep Medicine
2013	*Speech therapy proposals to the snoring patient^32^*	Journal of Communication Disorders [Portuguese]
2012	*Cephalometric analysis of modifications of the pharynx due to maxillo-mandibular advancement surgery in patients with obstructive sleep apnea^33^*	International Journal of Oral and Maxillofacial Surgery
2011	*Phonoaudiological assessment of patients with obstructive sleep apnea^34^*	Sleep Science
2011	*Swallowing dysfunction related to obstructive sleep apnea: a nasal fibroscopy pilot study^35^*	Sleep & Breathing
2010	*An interdisciplinary look at: obstructive sleep apnea^36^*	PET Informs (FOB / USP) [Portuguese]
2010	*Methods for increasing upper airway muscle tonus in treating obstructive sleep apnea: systematic review^37^*	Sleep & Breathing
2010	*Speech therapy and sleepy apnae: a review^38^*	CEFAC Journal [Portuguese]
2010	*Speech therapy and snore and sleep apnea^39^*	CEFAC Journal [Portuguese]
2009	*Effects of oropharyngeal exercises on patients with moderate obstructive sleep apnea syndrome^5^*	American Journal of Respiratory and Critical Care Medicine
2009	*Sleep obstructive apnea-hypoapnea syndrome and the phonoaudiological focus: literature review^40^*	CEFAC Journal [Portuguese]
2008	*Methods to increase muscle tonus of upper airway to treat snoring: systematic review^41^*	Neuro-Psychiatry archives [Portuguese]
2007	*Oral myofunctional therapy applied on two cases of severe obstructive sleep apnea syndrome^42^*	International Archives of Otorhinolaryngology
2007	*Speech therapy in the obstructive sleep apnea-hypopnea syndrome: case report^43^*	CEFAC Journal [Portuguese]
2006	*Auditory processing assessment in children with obstructive sleep apnea syndrome^44^*	Brazilian Journal of Otorhinolaryngology
2004	*Oral breathing and its relation to nocturnal snoring and obstructive sleep apnea syndrome^45^*	CoDAS
2004	*The relationship between sleep apnea, snoring and oral breathing^46^*	CoDAS
2004	*Treatment of obstructive sleep apnea syndrome (OSAS) through orthognatic surgery of mandibular advancement47*	Brazilian Journal of Orthodontics & Facial Orthopedics [Portuguese]
1999	*Alterations in soft tissue of oropharynx in obstructive sleep apnea patients^48^*	CoDAS

Legend: FOB/USP: Faculdade de Odontologia de Bauru USP.

Table 3 classifies by year, type of publication, and title of the 21 monographs, 13 dissertations, 8 theses, 1 book, and 8 book chapters that were included.

**Table 3:** Books, book chapters, monographs, dissertations, and thesis in the field of sleep disorders published by Brazilian SLPs, detailed by year of conclusion, type of study, and title.

Year	Type	Title
IN PROGRESS	Thesis	*Oral language in obstructive sleep apnea [Portuguese]^49^*
IN PROGRESS	Thesis	*Orofacial motor control in children with obstructive sleep apnea and primary snoring [Portuguese]^50^*
IN PROGRESS	Thesis	*Translation and cultural adaptation to Brazilian Portuguese of the quality of life questionnaire (SAQLI) to obstructive sleep apnea patients [Portuguese]^51^*
2017	Dissertation	*Characteristics of swallowing in individuals with obstructive sleep apnea syndrome [Portuguese]^52^*
2017	Dissertation	*Orofacial miofunctional study in children with obstructive sleep apnea [Portuguese]^53^*
2017	Book Chapter	*Speech, language and hearing sciences in sleep [Portuguese]^54^*
2016	Dissertation	*Effect of obstructive sleep apnea on adult hearing [Portuguese]^55^*
2016	Dissertation	*Evaluation of nasal geometry in adults with obstructive sleep apnea [Portuguese]^56^*
2016	Dissertation	*Functional characteristics of orofacial musculature in children with and without obstructive sleep apnea [Portuguese]^57^*
2016	Dissertation	*Long-latency auditory evoked potentials in obstructive sleep apnea [Portuguese]^58^*
2016	Monograph	*Contribution of phonoaudiology to the treatment of snoring in obstructive sleep apnea and hypopnea syndrome [Portuguese]^59^*
2016	Monograph	*Pharyngeal geometry pre and post myofunctional orofacial theraph in Individuals with obstructive sleep apnea [Portuguese]^60^*
2015	Thesis	*Effects of orofacial myofunctional therapy in patients with primary snoring and obstructive sleep apnea in the anatomy and function of the airway [Portuguese]^61^*
2015	Thesis	*Myofunctional orofacial and electromyographic evaluation of patients with obstructive sleep apnea [Portuguese]^62^*
2015	Dissertation	*Immediate effects of semi-fluid vocal tract exercises in patients with obstructive sleep apnea syndrome [Portuguese]^63^*
2015	Dissertation	*Rhinoometric characteristics and myofunctional assessment in individuals with obstructive sleep apnea syndrome who use CPAP [Portuguese]^64^*
2015	Monograph	*Elaboration of a phonoaudiological screening instrument for the treatment of snoring and OSAS [Portuguese]^65^*
2015	Monograph	*Myofunctional orofacial therapy in quality of life of women with obstructive sleep apnea syndrome [Portuguese]^66^*
2014	Thesis	*Effects of orofacial myofunctional therapy on snoring and sleep quality in patients with primary snoring and obstructive apnea in mild to moderate sleep [Portuguese]^67^*
2014	Dissertation	*Comparative analysis of swallowing after surgical treatment of OSAS: uvulopalatopharyngoplasty versus expander pharyngoplasty [Portuguese]^68^*
2014	Dissertation	*Evaluation of swallowing pattern in adult OSAS patients [Portuguese]^69^*
2014	Dissertation	*Young Doctor Project: health education actions aimed at obstructive sleep apnea and hypopnea syndrome [Portuguese]^70^*
2014	Monograph	*Systematic Review: the contribution of speech therapy in obstructive sleep apnea and hypopnea syndrome [Portuguese]^71^*
2014	Book chapter	*Evaluation of orofacial motricity in patients with obstructive sleep apnea syndrome^72^*
2014	Book chapter	*Noctural Breathing - from normality to alterations [Portuguese]^73^*
2014	Book chapter	*Snoring and obstructive sleep apnea [Portuguese]^74^*
2013	Monograph	*Effects of speech therapy on snoring [Portuguese]^75^*
2013	Monograph	*Speech-language pathology intervention in obstructive sleep apnea syndrome: state-of-the-art [Portuguese]^76^*
2012	Thesis	*Speech therapy as an adjunct to treatment with continuous positive airway pressure in patients with obstructive sleep apnea syndrome [Portuguese]^77^*
2012	Dissertation	*Oral and snoring breathing in childhood and adolescence: orofacial structures and musculature [Portuguese]^78^*
2012	Monograph	*Masticatory pattern of patients with obstructive sleep apnea [Portuguese]^79^*
2012	Monograph	*Quality of life in individuals with obstructive sleep apnea and hypopnea syndrome and snoring before and after speech therapy [Portuguese]^80^*
2011	Monograph	*Evaluation and comparison of language and pulsology in individuals with and without obstructive sleep apnea^81^*
2011	Monograph	*Myofunctional orofacial efficacy in the treatment of severe obstructive sleep apnea syndrome [Portuguese]^82^*
2011	Monograph	*Quality of life in adults with different degrees of obstructive sleep apnea [Portuguese]^83^*
2011	Monograph	*Speech-language therapy to the patient with apnea: case report [Portuguese]^84^*
2011	Monograph	*Tongue strength and swallowing pattern in adults with obstructive sleep apnea [Portuguese]^85^*
2011	Book chapter	*Obstructive sleep apnea syndrome [Portuguese]^86^*
2010	Monograph	*Investigation of speech-language pathology and audiology in obstructive sleep apnea syndrome in the city of Recife [Portuguese]^87^*
2009	Monograph	*Myofunctional therapy in obstructive sleep apnea correlated with surface electromyography data [Portuguese]^88^*
2009	Book	*Apnea and snoring: orofacial myofunctional treatment [Portuguese]^89^*
2008	Thesis	*Effects of oropharyngeal exercises in patients with moderate obstructive sleep apnea [Portuguese]^90^*
2008	Monograph	*Otorhinolaryngologists' knowledge about speech-language pathology in snoring [Portuguese]^91^*
2008	Monograph	*Sleep bruxism in oronasal breathing children 8 to 11 years of age: an association study [Portuguese]^92^*
2007	Book chapter	*Multifunction System (MFS) - advances in the treatment of OSAHS [Portuguese]^93^*
2007	Book chapter	*Oral respiratory and obstructive sleep apnea-hypopnea syndrome: the role of prevention and early treatment [Portuguese]^94^*
2007	Book chapter	*Roncopathy and OSAHS - phonoaudiological treatment rehabilitation of the pharyngeal dilator muscles [Portuguese]^95^*
2006	Monograph	*Relationship between myofunctional orofacial disorders and snoring in overweight patients [Portuguese]^96^*
2005	Dissertation	*Evaluation of the efficacy of speech-language intervention - myofunctional therapy - in snoring patients with and without obstructive sleep apnea syndrome [Portuguese]^97^*
2003	Monograph	*Characterization of snoring in individuals with and without obstructive sleep apnea hypopnea syndrome [Portuguese]^98^*
1999	Monograph	*Alterations in soft tissue of oropharynx in obstructive sleep apnea patients [Portuguese]^6^*

This search strategy resulted in the location of 214 participations in events of Brazilian SLPs, 63 lectures, and 151 presentations of scientific abstracts during the period from 2001 to 2017. The presentations and lectures occurred, in addition to Brazil, in eight other countries: Canada, the USA, Portugal, Spain, Chile, Peru, Colombia, and Argentina. Table 4 shows the year of the event, name, place of event, organizing institution, and name of the presentation/lecture.

**Table 4:** Scientific presentations and lectures of Brazilian SLPs at events, according to the year, name, place of the event, organizing institution, and name of the study/lecture.

Year	Event	Local	Organization	Scientific presentations (poster or oral communication)	Lectures
*2017*	17^th^ Paulista Congress of Pulmonology and Tisiology	São Paulo/SP- Brazil	SPPT Nov 15-18	-	· The multiple clinical faces of Sleep Apnea: Obstructive Sleep Apnea Phenotypes and Implications in treatment [Portuguese]^99^
*2017*	16^th^ Brazilian Congress of the Brazilian Society of Neuropsychology	São Paulo/SP- Brazil	SBN Nov 17-18	-	· Speech language and hearing sciences parameters and perspectives for the rehabilitation of sleep disorders^100^
*2017*	16^th^ Brazilian Congress of Sleep	Joinville/SP- Brazil	ABS, ABMS e ABROS Nov 1-4	· Application of imaging exams to speech-language pathology of sleep [Portuguese]^101^ · Excessive daytime sleepiness, insomnia and the influence of technologies on adolescents sleep [Portuguese]^102^ · Health actions during sleep week 2017 in the state of Sao Paulo: screening for obstructive sleep apnea [Portuguese]^103^ · Speech therapy treatment in OSA with snoring in Angle Class III patient - clinical case presentation [Portuguese]^104^ · Speech therapy treatment in OSA with snoring in a patient submitted to orthognathic surgery - clinical case presentation [Portuguese]^105^ · The influence of the use of communication technologies on teenagers sleep [Portuguese]^106^ · Translation and cultural adaptation to Brazilian Portuguese of the quality of life questionnaire (SAQLI) [Portuguese]^107^ · Tympanometric findings prior to adenotonsillectomy [Portuguese]^108^ · Tongue frenulum and sleep complaints: case report [Portuguese]^109^	· In addition to CPAP: alternatives for the treatment of sleep apnea - Which is the ideal patient for myofunctional therapy [Portuguese]^110^ · Impact of myofunctional therapy on CPAP adherence [Portuguese]^111^ · My child snore: functional rehabilitation of nasal breathing [Portuguese]^112^ · Reviewing the indications of intra-oral apparatus: Myofunctional aspects in the AIO candidate [Portuguese]^113^ · Sleep Apnea Phenotypes: Clinical [Portuguese]^114^ · Speech language and hearing sciences procedure in CPAP adherence in the elderly: a case report [Portuguese]^115^ · Speech language and hearing sciences therapy in the different approaches to OSA treatment [Portuguese]^116^ · Update on the treatment of sleep apnea beyond CPAP: with Myofunctional Therapy [Portuguese]^117^
*2017*	14^th^ World Sleep Congress	Prague, Czech Republic	World Sleep Society Oct 7-11	· Effectiveness of orofacial myofunctional therapy in obstructive sleep apnea in adults: systematic review^118^ · Effects of physical activity on quality of life and sleep quality in CPAP users^119^ · Electromyography analyses during DISE: applicability in hypoglossal nerve stimulation120 · Syntax consciousness and obstructive sleep apnea^121^ · Sleep awareness week 2017: actions of health promotion in the interior of São Paulo state^122^	· Myofunctional therapy for adult snoring and OSA: Changes in tongue fat and airway in a RCT^123^ · Pathways to update standards of care: How myofunctional therapy works in OSA^124^
*2017*	25^th^ Brazilian Congress of Speech language and hearing sciences	Salvador/BA- Brazil	SBFa Sep 12-15	*· Comparative Study of the Conservative and Surgical Treatments of Obstructive Sleep Apnea [Portuguese]^125^* · Effectiveness of orofacial myofunctional exercises in the treatment of snoring and sleep apnea: a case study [Portuguese]^126^ · Effectiveness of orofacial miofunctional therapy in obstructive sleep apnea in adults: systematic review [Portuguese]^127^ · Findings of videoendoscopy of deglutition in obstructive sleep apnea syndrome: Case report [Portuguese]^128^ · Language and aspects of premature children’s sleep [Portuguese]^129^ · Obesity: risk factor for obstructive sleep apnea [Portuguese]^130^ *· Obstructive Sleep Apnea Syndrome and swallowing: oral myofunctional, respiratory and neuromuscular coordination aspects [Portuguese]^131^* · Perception of the quality of life of patients with obstructive sleep apnea syndrome [Portuguese]^132^ · Sleep disorders: a proposal of health education for the elderly [Portuguese]^133^ · The sleep quality of the patient that snore [Portuguese]^134^	· Practices of orofacial motricity in respiratory sleep disorders [Portuguese]^135^
*2017*	15^th^ Academic Seminary of the Speech language and hearing sciences Course	São Paulo/SP- Brazil Sep 4-5	FCMSCSP		· Changes in sleep in orofacial motricity [Portuguese]^136^
*2017*	24^th^ COFAB	Bauru/SP- Brazil	Department of Speech-language and hearing sciences - USP-FOB Aug 16-19	*· Deleterious oral habits in children with respiratory complaints during sleep [Portuguese]^137^* *· Obstructive sleep apnea and metasynthetic ability [Portuguese]^139^* *· Obstructive sleep apnea in young adults with pharyngeal flap [Portuguese]^138^* *· Participation of Brazilian Speech language and hearing sciences in Sleep Science [Portuguese]^140^* *· Sleep Week 2017: health promotion in the interior of São Paulo [Portuguese]^141^* *· Tongue function in patients with Obstructive Sleep Apnea Syndrome [Portuguese]^142^*	-
*2017*	2^nd^ Ibero-American Orofacial Motricity Symposium	Madrid- Spain	University Complutense of Madrid and Institute EPAP Jun 16-17	-	*· Stomatognathic alterations, OSAS and phonoaudiological intervention in interdisciplinary approach [Spanish]^143^*
*2017*	10^th^ Brazilian meeting of Orofacial Motricity	Belo Horizonte- Brazil	ABRAMO Jun 1-3	· Myofunctional orofacial aspects in patients with obstructive sleep apnea syndrome [Portuguese]^144^ · OSAS in childhood [Portuguese]^145^ · Speech language and hearing sciences in obstructive sleep apnea: literature review^146 ^· Speech language and hearing sciences performance in obstructive sleep apnea and hypopnea syndrome: case report^147^	· Speech language and hearing sciences view: performance in snoring and sleep apnea^148^
*2017*	15^th^ Speech language and hearing sciences week	Campinas/SP-Brazil	UNICAMP May 22-26	-	*· Speech language and hearing sciences in sleep: performance in snoring and sleep apnea^149^*
*2017*	15th Academic Journal of Speech language and hearing sciences	Belo Horizonte- Brazil	PUC May 18-19	-	· Interdisciplinary action and respiratory sleep disorders [Portuguese]^150^
*2017*	3^rd^ International Congress of Speech-language and hearing sciences and Meeting of Latin American Integration	Rosario- Argentina	National University of Rosario May 10-13	-	· Advances in Orofacial Motricity: “sleep disorders” [Spanish]^151^
*2017*	2^nd^ AAMS Congress	Chicago - USA	AAMS March 1-5	-	· Different approaches of Orofacial Myofunctional Therapy (OMT) in OSA patients: Isolated OMT or within interdisciplinary treatments (CPAP; Intraoral Appliances; Hypoglossal Nerve Stimulation)^152^
*2016*	15^th^ Brazilian Congress of Sleep	São Paulo/SP-Brazil	ABS, ABMS and ABROS	AMOS: orofacial myofunctional evaluation for obstructive sleep apnea [Portuguese]^153^ Case report: Speech language and hearing sciences intervention in oropharyngeal hypofunction: case report [Portuguese]^154^ Case report: Speech language and hearing sciences treatment of obstructive sleep apnea with snoring: a case report [Portuguese]^155^ Correlation enters the oropharyngeal cross-sectional area and the severity of obstructive sleep apnea [Portuguese]^156^ Importance of the anamnestic data of snoring complaint in children with adapted deglutition in the initial phase of mixed dentition [Portuguese]^157 ^Unsupervised polysomnography in children [Portuguese]^158^ Obesity and Sleep Respiratory Disorders: quantitative analysis and associations [Portuguese]^159^ Obstructive sleep apnea in young adults with cleft lip and palate is related to maxillomandibular discrepancies? [Portuguese]^160^ REM-sleep apnea-hypopnea index predominance in subjects with cleft lip and palate and obstructive sleep apnea [Portuguese]^161^ Respiratory symptoms and nasalance of speech after resection of the pharyngeal flap [Portuguese]^162^	· How to select the patient: Speech-language pathology [Portuguese^169 ^· Multidisciplinary Treatment of Obstructive Sleep Apnea (OSA) based on clinical cases [Portuguese^170^ · Orofacial myofunctional evaluation [Portuguese^171^ · Phenotype of the patient with OSA [Portuguese^172^ · Post-surgical speech language hearing sciences rehabilitation [Portuguese^173^
*2016*				*Respiratory sleep disorders in young and middle-aged adults with cleft lip and palate assessed by validated questionnaires and nocturnal polysomnography: effect of age [Portuguese]^163^* Sleep quality questionnaires for the pediatric population used in Brazil [Portuguese]^164^ Speech and Hearing Intervention in Oropharyngeal Hypofunction: Case Report [Portuguese]^165^ Undervised polysomnography helps planning adenoamigdalectomy? [Portuguese]^166^ Week of sleep and speech language and hearing sciences: dissemination and awareness about sleep in Brazil [Portuguese]^167^ What are the role of questionnaires? Berlin and Epworth in the research of the association of chronic obstructive pulmonary disease and syndrome of obstructive sleep apnea [Portuguese]^168^	
*2016*	18^th^ International Congress of Odontology of BAHIA/10^th^ Speech language and hearing sciences Seminary	Salvador/BA- Brazil	ABO- BA Nov 2016	-	*· Speech language and hearing sciences evaluation and treatment in Obstructive Sleep Apnea [Portuguese]^174^*
*2016*	24^th^ Brazilian Congress of Speech language and hearing sciences	São Paulo/SP- Brazil	SBFa Oct 2016	· Food intake of patients with obstructive sleep apnea: literature review^175^ · Orofacial miofunctional characteristics in patients with snoring and obstructive sleep apnea^176^ · Oromiofunctional therapy as adjuvant to the treatment of upper airway resistance syndrome: a case report^177^ · Protocol of evaluation for obstructive sleep apnea: systematic review178 · Relationship between obesity and sleep psychic aspects^179^ · Scientific production on snoring: analysis of Brazilian speech language hearing sciences^180^	*Interdisciplinary symposium -diagnosis and treatment of sleep disorders: Speech language hearing sciences approach [Portuguese]^184^*
				· Speech language and hearing sciences assistance in cases of apnea in the city of João Pessoa^181^ · Speech language hearing sciences therapy and therapeutic adherence in obstructive sleep apnea syndrome^182^ · Study of snoring in healthy elderly^183^	
*2016*	2^nd^ Ibero-American Orofacial Motricity and3rd American Meeting	Santa Marta- Colombia	CMOL Sep 15-17	-	Interdisciplinary treatment of obstructive sleep apnea syndrome [Spanish]^185^
*2016*	10^th^ Congress of ALAT	Santiago - Chile	ALAT Jul 6-9	· Insertion and contribution of speech-language pathology in sleep disorders [Spanish]^186^ · Obstructive sleep apnea and oral language in children [Spanish] ^187^ · Sleep quality in children: differences in the questionnaires used in Brazil [Spanish]^188^ · Sleep quality in children: review of the questionnaires used in Brazil [Spanish]^189^ · Unassisted childhood polysomnography [Spanish]^190 ^	-
*2016*	Meeting of Orofacial Motricity	Bauru/SP- Brazil	ABRAMO Jun 3-4	· Comparative analysis of respiratory symptoms related to sleep in children with cleft lip and palate and pharyngeal flap [Portuguese]^191^ · Production of brazilian speech therapist in sleep disorders area [Portuguese]^192^ · Relationship between respiratory symptons and nasalance of talk in young adults with cleft lip palate fissure^193^ · Self-reported orofacial myofunctional aspects of freshmen undergraduate students in Speech Language Pathology and Audiology at UFSC [Portuguese] ^194^	*Obstructive sleep apnea: interdisciplinary approach [Portuguese]^195^*
*2016*	2^th^ Congress of speech language and hearing sciences of the Faculty of Medicine of UFMG	Belo Horizonte, Brazil	UFMG May 19-21	-	Speech language and hearing sciences treatment on respiratory sleep disorders [Portuguese]^196^
*2016*	9^th^ Postgraduate meeting of UNESP	Botucatu/SP- Brazil	UNESP-FMB May 18-19	· Obstructive sleep apnea and oral language [Portuguese]^197^	-
*2016*	1^st^ International Congress of Portuguese Society of Speech Therapy	Santo Tirso- Portugal	SPTF May 13-15	-	· Interfaces of speech and hearing pathology in orofacial motor: new areas of intervention (OSA)^198^*· Obstructive sleep apnea: evidence of orofacial myofunctional therapy [Portuguese]^199^*
*2016*	7^th^ International Symposium - Surgery Sleep and Breathing	São Paulo/SP- Brazil	ISSS and FORL Apr 15-16	· Obstructive Sleep Apnea and oral language: Sleep quality in children [Portuguese]^200^	*Effects of oropharyngeal exercises on snoring and OSA [Portuguese]^201^*
*2016*	4^th^ Brazilian Congress of Electromyography and Kinesiology	Ribeirão Preto/SP- Brazil	University of São Paulo-Ribeirão Preto Apr 14-16	· Electromyographic activity of masseter and temporal muscles in patients with obstructive sleep apnea [Portuguese]^202^	
*2015*	Event in commemoration of the Speech-Language Pathologist's Day "Speech-language pathology - a promising area"	Recife/PE-Brazil	CRFa 4^th^ region Dez 10	-	*Speech language hearing sciences in the treatment of Obstructive Sleep Apnea and Snoring [Portuguese]^203^*
*2015*	14th International Symposium on Sleep and Breathing	Porto de Galinhas/PE- Brazil	ISSS and FORL Oct 15-28	-	*Effects of myofunctional therapy in patients with primary snoring and obstructive sleep apnea on upper airway anatomy by MRI [Portuguese]204*
*2015*	15^th^ Brazilian Congress of Sleep	Porto de Galinhas/PE-Brazil	ABS, ABMS and ABROS Oct 28-31	· A transdisciplinary approach to OSAHS in the speech language and hearing sciences clinic: a case study [Portuguese]^205^ · Children with language impairment: deleterious oral habits and sleep complaints [Portuguese]^206^ · Scientific publications on speech language and hearing sciences aimed at obstructive sleep apnea [Portuguese]^^207^^ · Speech language and hearing sciences therapy applied to four cases with obstructive sleep apnea syndrome [Portuguese]208 · Speech-language and hearing sciences therapy for the treatment of OSAHS [Portuguese]^209^	*· “Update on Sleep-disordered breathing” with five subtopics, including “Speech language and hearing sciences and OSA - discussing the evidence in the evaluation and clinical practice” [Portuguese]^210^* *· Interdisciplinary symposium: “Treatment of obstructive sleep apnea: beyond CPAP” with the lecture “Myofunctional exercises” [Portuguese]^211^*
*2015*	23^th^ Brazilian Congress of Speech language and hearing sciences and 9^th^ International Congress of Speech and Hearing Therapy-	Salvador/BA-Brazil	SBFa Oct 14-16	· Correlation between degree of daytime sleepiness and nasal volume in OSA patients: a pilot study [Portuguese]^212^	· *Interdepartmental symposium 1: the choice of exercises in orofacial myofunctional disorders: snoring, apnea and facial paralysis [Portuguese]^216^*
				· Orofacial myofunctional therapy in the quality of life of women with obstructive sleep apnea syndrome [Portuguese]^213^ · Snoring and quality of life in young adults [Portuguese]^214^ · Speech-language pathology in snoring: a case report [Portuguese]^215^	· *Interdepartmental symposium 2: obstructive sleep apnea, orofacial motricity and dysphagia: What is there in common? Orofacial motricity [Portuguese]^217^*
*2015*	44^th^ International Association of Orofacial Myology Congress	Lake Buena Vista, USA	IAOM	· *Breathing and Sleep Quality in Children218*	
*2015*	AAMS 1st Congress	Los Angeles -USA	AAMS Sep 9-13	-	· Challenges of interdisciplinarity in OSA, in TMD and in orthognathic surgery rehabilitation^219^ · Sleep-disordered breathing and myofunctional therapy: where do we go from here?^220^
*2015*	COFAB 22^th ^Speech language annguage and hearing sciences Congress of Bauru	Bauru/SP - Brazil	Department of Speech-Language Pathology- FOB/USP Aug 26-29	*· Cancer and Obstructive sleep apnea [Portuguese]^221^* · Deleterial and delayed habits and sickness related to sleep in children with language change^222^ · * Level of evidence and area of speech therapy in scientific publications on obstructive sleep apnea [Portuguese]^223^*	· The Speech Pathology Intervention in Obstructive Sleep Apnea [Portuguese]^224^
*2015*	1st American Encounter and 2nd Ibero-American Meeting of Orofacial Motricity	Lima - Peru	CMOL Jun 26-27	-	*· Sleep Disorders: myofunctional approach and orofacial structural relations [Spanish]^225^*
*2015*	3^rd^ Journey of Orthognathic Surgery and Orthodontics	São Paulo/ SP- Brazil	IEP/HSL May 23-24	-	*· Snoring and sleep apnea: a multiprofessional approach. Speech therapy in OSAS [Portuguese]^226^*
*2015*	American Thoracic Society International Conference	Denver-USA	ATS May 15-20	*· Effects of Oropharyngeal Exercises on Snoring: A Randomized Trial^227^*	-
*2015*	8^th^ Brazilian Meeting of Orofacial Motricity	João Pessoa /PE- Brazil	ABRAMO May 15-16	· Has cleft type an effect on pharyngeal flap outcomes?^228^ · *Speech Therapy contributions in Obstructive Sleep Apnea Syndrome: An Integrative Review^229^* · The use of acoustic pharyngometry in the diagnosis of obstructive sleep apnea: a systematic review [Portuguese]^230 ^· The use of acoustic rhinometry in the diagnosis of obstructive sleep apnea: a systematic review [Portuguese]^231^	
*2015*	1^st^ Ibero-American Symposium on Orofacial Motricity	Porto - Portugal	*AAMS, ABRAMO IAOM, SPTF, SBFa*, General Council of Speech-language Colleges Jan 30 fev 01	-	*Orofacial myofunctional intervention in OSA and snoring [Portuguese]^232^*
*2014*	23^th^ Brazilian Congress of Speech language and hearing sciences	Joinville/SC - Brazil	SBFa Oct 8-11	· Effect of Speech-Language therapy for patients with obstructive sleep apnea syndrome [Portuguese]^233^ · Influence of Speech language hearing sciences treatment on the quality of life of individuals with OSAS [Portuguese]^234^ · Rhinomanometric characteristics and nasal aeration in individuals with OSAHS treated with CPAP: an evaluation proposal [Portuguese]^235^	· Snoring and OSAS: how to treat? [Portuguese]^236^ · OSAS: speech language hearing and sciences procedure [Portuguese]^237^
*2014*	7th Brazilian Meeting of Orofacial Motricity	São Paulo/SP - Brazil	ABRAMO May 16-17	*Nasopharyngeal dimensions in subjects with or without pharyngeal flap238*	· Panel discussion on snoring and apnea *[Portuguese]239*
*2014*	21^th^ Speech language and hearing sciences Journey of Bauru	Bauru/SP - Brazil	USP-FOB	· Obstructive sleep apnea in legislation [Portuguese]^240^ · Sleep disturbances and behavioral profile in individuals with complaints of language *disorders [Portuguese]^241^*	· Workshop: Speech language and hearing sciences for Snoring and Apnea [Portuguese]^242^
*2014*	1^st^ Speech language and hearing sciences symposium applied to Sleep	São Paulo/SP - Brazil	ABS	-	· Speech language and hearing sciences assessment in Obstructive Sleep Apnea [Portuguese]^243^*· Speech language and hearing sciences treatment in snoring and obstructive sleep apnea [Portuguese]^244^*
*2014*	22^th^ Annual Meeting of the Spanish Sleep Society	San Sebastian - Spain	Spanish Sleep Society	-	· Orofacial miofunctional therapy for obstructive sleep apnea and snoring [Spanish]^245^
*2013*	11^th^ Congress of Sao Paulo of Sleep Medicine	São Paulo/SP -Brazil	APM and AMB	-	· *Evaluation and Speech Pathology approach in OSAS [Portuguese]^246^*
*2013*	5^th^ World Congress on Sleep Medicine	Valencia-Spain	WASM	· Analysis of validity in adults of the expanded protocol of orofacial myofunctional evaluation with scores^247^ · *Assessment of surface EMG supra-hyoid muscle activity in apneic patients compared to healthy subjects. A pilot study^248^*	-
				· *Electronic tutor about obstructive sleep apnea syndrome: development and evaluation of a health collaborative network^249^· Orofacial myofunctional evaluation with scores in subjects with obstructive sleep apnea250· Orofacial myology therapy: a case report of upper airway resistance syndrome.^251^· Predictors of uvulopalatopharyngoplasty success in the treatment of obstructive sleep apnea syndrome^252^*	
*2013*	International Pediatric Sleep Association Congress	Porto Alegre/RS - Brazil	IPSA	*· Working memory, lexical access and sleep quality in children [Portuguese]^253^* *· Young Doctor Project: health educational actions focused on obstructive sleep apnea [Portuguese]^254^*	· A call for newborn frenum inspections in sleep disorder prevention^255^
*2013*	VI Brazilian Meeting of Orofacial Motricity	Fortaleza/CE- Brazil	ABRAMO	*· Health promotion in obstructive sleep apnea syndrome: literature review [Portuguese]^256^* *· The ability of OMES-E to discriminate the orofacial condition of healthy adults with Obstructive Sleep Apnea and association between variables [Portuguese]^257^*	*· Evaluation of Snoring and Obstructive Sleep Apnea [Portuguese]^258^*
*2013*	28º International Meeting of audiology	Salvador/BA-Brazil	ABA	· Auditory alterations and sleep disorders: literature review [Portuguese]^259^	-
*2013*	21º Brazilian Congresso of Speech-language pathology and 2^nd^ Ibero-American of Speech-language Pathology	Porto de Galinhas/PE- Brazil	SBFa	· Construction of a questionnaire estimating the level of knowledge about obstructive sleep apnea syndrome [Portuguese]^260^ · Obstructive sleep apnea syndrome and oropharyngeal dysphagia: clinical case [Portuguese]^261^ · Speech-language pathology intervention in obstructive sleep apnea syndrome: state-of-the-art [Portuguese]^262^	-
*2013*	13^th^ International Symposium on Sleep and Breathing	Montreal-Canada	ISSS e FORL Foundation of Otolaryngologist	· Tongue fatigability is associated to pharyngeal closing pressure^263^	-
*2013*	29^th^ World Congress of the IALP	Turin - Canada	IALP	· Effect of speech therapy as adjunct treatment to CPAP, on the quality of life of patients with obstructive sleep apnea^264^ · Oropharyngeal exercises improved adherence to continuous positive airway pressure treatment^265^	
*2013*	International Association of Orofacial Myology	Orlando -USA		· Moment of therapeutical discharge of the patient with sleep disorder^266^	-
*2013*	43º Brazilian Congress of Otolaryngology and Cervical-Facial	São Paulo/SP- Brazil	ABORL-CCF	-	*· What should be done when there is no surgery for snoring and apnea? Panel discussion: Who benefits from myofunctional therapy? [Portuguese]^267^*
*2012*	XX Brazilian Congress of Speech language and hearing sciences	Brasília/DF - Brazil	SBFa	· Analysis of the national production on Obstructive Sleep Apnea Syndrome in a speech pathology perspective [Portuguese]^268^ · Chewing pattern of patients with Obstructive Sleep Apnea Syndrome [Portuguese]^269^ · Myofunctional orofacial evaluation in subjects with obstructive sleep apnea [Portuguese]^270^ · Oral breathing is associated with obstructive sleep apnea severity [Portuguese]^,^	-
*2012*	Bauru Journey of Speech language and hearing sciences	Bauru/SP - Brazil	USP- FOB	· The maximum isometric tongue strength and the classification of mallampati in individuals with obstructive sleep apnea syndrome [Portuguese]^272^	-
*2012*	V Brazilian Meeting of Orofacial Motricity	Curitiba/PR- Brazil	ABRAMO	-	*· Snoring and apnea - How do I treat? [Portuguese]^273^*
*2011*	4^th^ International Congress of the Association of Sleep Medicine	Quebec/Canada	WASM	*· Oropharyngeal exercises as therapy of obstructive sleep apnea in a patient with chronic obstructive pulmonary disease^274^*	-
*2011*	19° Brazilian Congress of Speech language and hearing sciences and 8º International congress of Speech language and hearing sciences	São Paulo/SP- Brazil	SBFa	*· Apnea and Speech-language pathology: Proposal for pharyngeal stimulation [Portuguese]^275^* · Electromyographic characterization of deglutition in individuals with and without clinical swallowing changes [Portuguese]^276^ *· Evaluation of chewing on patients with obstructive sleep apnea syndrome [Portuguese]^277^· Speech-language pathology indication in obstructive sleep apnea and hypopnea syndrome in the city of Recife [Portuguese]^278^· Tongue strength and swallowing pattern in men with obstructive sleep apnea [Portuguese]^279^*	*· Speech Pathology therapy in obstructive sleep apnea syndrome [Portuguese]^280^*
*2011*	XIII Brazilian Congress of Sleep	Belo Horizonte - Brazil	ABS	-	· Speech-language pathology therapy in obstructive sleep apnea syndrome [Portuguese]^281^
*2011*	VIII Sleep Medicine Congress of São Paulo	São Paulo/SP- Brazil	APM e AMB	-	*· Speech-language therapy in OSA [Portuguese]^282^*
*2011*	XXXVII Week of Studies of the Faculty of Speech language and hearing sciences at the Center for Life Sciences	Campinas/SP- Brazil	PUC-Campinas	-	*· Snoring and apnea [Portuguese]^283^*
*2011*	IV Brazilian Meeting of Orofacial Motricity	Natal/RN-Brazil	ABRAMO	*· Obstructive sleep apnea syndrome: etiology, pathophysiology and speech-language pathology intervention [Portuguese]^284^*	-
*2010*	XVI Annual Scientific Meeting	Poconé/MT-Brazil	ABFCOC	-	*· Oral breathing - snoring and apnea [Portuguese]^285^*
*2010*	18º Brazilian Congress of Speech-language hearing sciences	Curitiba/PR-Brazil	SBFa	· Effect of the sound blowing exercise on the voice of the elderly [Portuguese]^286^ · Indicative of obstructive sleep apnea syndrome in military police hospital staff [Portuguese]^287^	-
				· Proposal of a protocol for the quantitative evaluation of the posterior region of the mouth before and after speech therapy in subjects with OSAHS and snoring [Portuguese]^288^*· Speech-language pathology performance in obstructive sleep apnea and hypopnea syndrome: what the literature says [Portuguese]^289^*	-
*2010*	20^th^ Congress of the European Sleep Research Society	Lisboa - Portugal	ESRS	· *Assessment of orofacial motricity in obstructive sleep apnea patients: a comparison between patients with and without nasal alterations. [Portuguese]^290^*	-
*2010*	II Congress of Speech language and hearing sciences hospital	Belém/PA- Brazil	Tuiuti University of Parana	*· Speech-language pathology actuation in snoring and Sleep Apnea [Portuguese]^291^*	-
*2009*	17º Brazilian Congress of Speech language and hearing sciences and 1º Ibero-American Speech language hearing sciences Congress	Salvador/BA- Brazil	SBFa	*· Analysis of tongue and soft palate evaluation in patients with obstructive sleep apnea syndrome [Portuguese]^292^* *· Oral myofunctional therapy in patients with severe obstructive sleep apnea [Portuguese]^293^* *· Sleep bruxism in oronasal breathing children 8 to 11 years of age: an association study [Portuguese]^294^* *· Speech-language pathology treatment in Obstructive Sleep Apnea and Hypopnea Syndrome: Clinical case report [Portuguese]^295^*	-
*2009*	3º World Sleep Congress	São Paulo/SP - Brazil	WASM	· Phonoaudiological assessment of patients with obstructive sleep apnea [Portuguese]^296^	-
*2008*	16º Brazilian Congress of Speech language and hearing sciences	Campos do Jordão/SP - Brazil	SBFa	· Acting of brazilian speech therapist together with patients with obstructive sleep apnea syndrome (OSAS) [Portuguese]^297^ · *Descriptive analysis of anthropometric measures (MA) in patients with obstructive sleep apnea syndrome (OSAS) [Portuguese]^298^*	-
				*· Evaluation of the orofacial motricity and the impact on the quality of life of individuals with Obstructive Sleep Apnea and Hypopnea Syndrome [Portuguese]^299^* *· Speech-language pathology in patients with obstructive sleep apnea syndrome [Portuguese]^300^*	
*2007*	2º Composium Internacional Association of Logopedics and Phoniatrics	São Paulo/SP- Brazil	IALP	*· Obstructive Sleep Apnea-Hypopnea Syndrome: orofacial motricity [Portuguese]^301^*	-
*2007*	XV Brazilian Congress of Speech language and hearing sciences	Gramado - Brazil	SBFª	· Acting of brazilian speech therapist in obstructive sleep apnea syndrome: case report [Portuguese]^302^	
*2006*	5º FORL Foundation of Otolaryngologist Congress	São Paulo	FORL	-	Current Approaches: Speech-language therapy in snoring^303^
*2005*	X Brazilian Congress of Sleep	Curitiba/PR- Brazil	ABS	*· Acoustic characterization of snoring in different rates of obstructive sleep apnea and hypopnea [Portuguese]^304^*	-
*2005*	XIII Brazilian Congress of Speech language and hearing sciences	Santos/SP- Brazil	SBFa	*· Evaluation of the stomatognathic system in patients with obstructive sleep apnea and hypopnea syndrome [Portuguese]^305^*	-
*2004*	XII Brazilian Congress of Speech language and hearing sciences	Foz do Iguaçu/PR - Brazil	SBFa	*· Characterization of snoring in individuals with and without obstructive sleep apnea-hypopnea syndrome [Portuguese]^306^*	
*2004*	3^rd^ Annual Symposium: Care of the Professional Voice	Filadélfia/PE-USA	The Voice Foundation	*· Characterization of snoring in people with Obstructive Sleep Apnea Syndrome [Portuguese]^307^*	-
*2003*	IX Journey of Speech language and hearing sciences	Marília/SP - Brazil	UNESP-Marília	· Speech-language pathology attendance in a case of obstructive sleep apnea submitted to orthognathic surgery [Portuguese]^308^	-
*2003*	V International Congress of Speech-language pathology, XI Brazilian Congress of Speech-language pathology	Fortaleza/CE- Brazil	SBFa	*· Characterization of snoring in individuals with and without obstructive sleep apnea-hypopnea syndrome [Portuguese]^309^*	-
*2003*	IX Brazilian Congress of Sleep	Vitória/ES- Brazil	ABS	*· Speech-language pathology intervention in the snoring patient [Portuguese]^310^*	-
*2003*	APSS 17^th^ Annual Meeting	Pection/BC-Canada	APSS	· Phonoaudiological work at obstructive sleep apnea [Portuguese]^311^	-
*2001*	VIII Brazilian Congress of Sleep	Salvador/BA-Brazil	ABS	*· Speech-language pathology work in patients who snore [Portuguese]^312^*	-

Legend: **AAMS** : Academy of Applied Myofunctional Sciences; **ABA** : Brazilian Academy of Audiology; **ABFCOC** : Brazilian Academy of Pathophysiology Cranio-oro-cervical; **ABMS** : Brazilian Association of Sleep Medicine; ABO- BA Brazilian Association of Dentistry- Bahia; **ABORL-CCF** : Brazilian Association of Otolaryngology and Cervical-Facial Surgery; **ABRAMO** : Brazilian Association of Orofacial Motricity; **ABROS** : Brazilian Association of Sleep Odontology; ** ABS** : Brazilian Association of Sleep; **ALAT** : Latin-American Association of Thorax; **AMB** : Brazilian Medical Association; **APM** : Paulista Association of Medicine; **APSS** : Associated Professional of Sleep Societies; **ATS** : American Thoracic Society; **CMOL** : Latin-American Orofacial Motricity Community; **CRF^a^** : Regional Speech and hearing Counseling; **COFAB** : Speech language and hearing sciences Congress of Bauru; **ESRS** : European Sleep Research Society; **FCMSCSP** : Faculty of Medical Sciences of Santa Casa of Sao Paulo; FORL: Foundation of Otolaryngologist; IALP: International Association of Logopedics and Phoniatrics; IAOM: International Association of Orofacial Myology; **IEP/HSL** : Institute of Teaching and Research /Syrian-Lebanese Hospital; Institute **EPAP** : Institute of Education, Employability and Health; ** IPSA** : International Pediatric Sleep Association; **ISSS** : International Surgical Sleep Society; **PUC** : Pontificial University Catholic- Campinas; **PUC** : Pontifical Catholic University; **SBF^a^** : Brazilian Society of Speech language and hearing sciences; **SBN** : Brazilian Society of Neuropsychology SPPT: Paulista Society of Pulmonology and Tisiology; **SPTF** : Portuguese Society of Speech Therapy; **UNESPFMB** : Faculty of Medicine of Botucatu Paulista State University; UFMG Federal University of Minas Gerais; **UNICAMP** : Campinas State University; **USP-FOB** : University of São Paulo-Faculty of Dentistry of Bauru; **WASM** : World Association of Sleep Medicine.

In total, the authors found 97 scientific presentations, 41 lectures, 40 papers, 12 monographs, 12 dissertations, 8 thesis, 8 book chapters, and 1 book, specified by year, as shown in Table 5.

**Table 5:** List of scientific presentations, lectures, articles, monographs, dissertations, thesis, book chapters, and books by Brazilian SLPs in the area of sleep disorders, according to the year of publication (in ascending order).

*Year*	Articles	Monographs	Dissertations	Thesis	Book chapters	Book	Scientific presentations	Lectures	TOTAL/year
*1999*	1	1	-	-	-	-	-	-	2
*2000*	-	-	-	-	-	-	-	-	-
*2001*	-	-	-	-	-	-	1	-	1
*2002*	-	-	-	-	-	-	-	-	-
*2003*	-	1	-	-	-	-	4	-	5
*2004*	3	-	-	-	-	-	2	-	5
*2005*	-	-	1	-	-	-	2	-	3
*2006*	1	1	-	-	-	-	-	1	3
*2007*	2	-	-	-	3	-	2	-	7
*2008*	1	2	-	1	-	-	4	-	8
*2009*	2	1	-	-	-	1	5	-	9
*2010*	4	1	-	-	-	-	6	1	12
*2011*	2	5	-	-	1	-	7	4	19
*2012*	1	2	1	1	-	-	5	1	11
*2013*	3	2	-	-	-	-	18	4	27
*2014*	4	1	3	1	3	-	6	7	25
*2015*	5	2	2	2	-	-	18	12	41
*2016*	6	2	4	-	-	-	37	13	62
*2017*	5	-	2	-	1	-	34	20	62
*In progress*	-	-	-	3	-	-	-	-	3
*TOTAL/type*	**40**	**21**	**13**	**8**	**8**	**1**	**151**	**63**	**305**

## DISCUSSION

SLHS involves activity in the prevention, assessment, diagnosis, and treatment of speech, language, social communication, cognitive communication, and swallowing disorders in children and adults^313^. Therefore, performance in sleep disorders is based on the interface of the areas of competence, especially concerning voice, breathing, chewing, swallowing, and human communication in general. Any performance should be based on scientific evidence^314^. Thus, the authors reviewed the participation of Brazilian SLPs in treatment of sleep disorders, targeting the best benefits for the patient^315^.

Among the 40 articles found, only 12 of them were published in journals that presented impact factor, a fact that is related to the low citation index of the journals focused on SLHS^316^ (Figure 1, Table 1, and Table 2).

Carrying out randomized studies and publication in a journal with high international impact resulted in greater interest for further investigations in scientific research in Brazil, with the development of techniques of orofacial myofunctional therapy in moderate OSA^5^. In 2015, a systematic review and meta-analysis^317^ showed that from a total of nine studies involving outcomes with reported polysomnography and sleepiness of adult patients, four of these studies were by Brazilians.

The publication of a randomized trial of techniques of orofacial myofunctional therapy in moderate OSA in an international journal with high impact encouraged Brazilian SLPs to conduct further scientific research in this area^5^. A high number of dissertations realized in public federal or state institutions were published in the years 2012, 2014, and 2015 (see Table 3). Therefore, a systematic review and meta-analysis of the outcomes of myofunctional therapy in obstructive breathing disorders during sleep in adults, published in 2015^317^, showed that from a total of nine studies, four were conducted by Brazilian SLPs.

As highlighted in Table 4, the lectures and presentations occurred at events organized by national and international associations/societies with strong scientific participation in the areas of both SLHS and sleep. The most frequent location of the events was Brazil, but we found participation in eight other countries (two from Europe, two from North America, and four from South America).

The number of scientific productions in this area more than doubled from 2010 to 2011; however, the highest production rates were observed in 2015 and 2016 (Table 5). Certainly, the advance in the knowledge of sleep medicine and its widespread dissemination in the media are notorious and are generating the interest of health professionals from all areas, including SLPs.

The search strategy of this article was not designed to provide any systematic or comprehensive results, but rather to provide an overview of the Brazilian contribution to the field. Future research employing a proper systematic review methodology is warranted in order to gather together and summarize all the available evidence in a broad a comprehensive manner.

There are innumerable public health problems related to sleep disorders, affecting occupational cases^1^^-^^3^ and interfering in everyday life^318^^,^^319^. Although the most frequent SLP approach refers to sleep-breathing disorders, we may note a growing interest in the understanding of the relationship of sleep disorders with other complaints and disorders, such as attention deficit disorder, written language disorders, hearing loss, and so on.^19^

The efficacy of orofacial myofunctional therapy is still questioned, and SLPs’ interventions are not widely recognized nationally or internationally. The main reason for this is the lack of randomized studies that evaluate its efficacy for the treatment of sleep-disordered breathing, for improvement of the quality of life, and/or for the improvement of adherence to CPAP.^17^ Likewise, most published studies are recent and present transversal evaluations or short-term follow-up results. There is a need for long-term follow-up studies and posterior reviews to enhance the evidence; thus, studies of the performance of SLPs in sleep disorder treatment should be encouraged.

## CONCLUSIONS

Brazilian SLHS shows a pioneering approach in the performance of SLPs in diagnosis and treatment of sleep disorders, mainly obstructive breathing disorders during sleep. This statement is supported by the increasing number of scientific publications in the formats of articles in national and international journals, monographs, thesis, dissertations, books, and abstracts in annals of events, most of these at interdisciplinary events of national and international scope. Scientific articles are published in journals of the area of SLHS, thus remaining with a lower dissemination. Efforts to improve the publications in multidisciplinary journals with a higher impact factor should be made.
